# Clinical Use of FSH in Male Infertility

**DOI:** 10.3389/fendo.2019.00322

**Published:** 2019-05-24

**Authors:** Hermann M. Behre

**Affiliations:** Center for Reproductive Medicine and Andrology, University Hospital Halle, Martin Luther University Halle-Wittenberg, Halle, Germany

**Keywords:** FSH, hMG, hCG, male infertiltiy, hypogonadotropic hypogonadism, idiopathic male infertility

## Abstract

The established clinical indication for FSH use in male infertility is the treatment of patients with hypogonadotropic hypogonadism for stimulation of spermatogenesis that allows the induction of a clinical pregnancy in the female partner and finally the birth of a healthy child. Several clinical studies with urinary, purified, and recombinant FSH preparations in combination with hCG have demonstrated the high treatment efficacy regarding these clinical endpoints. Shortcomings of this hormone therapy are the long duration of treatment, sometimes longer than 2 years, and the inconvenience of injections every second or third day. However, improvements of therapy might be expected with new hormonal treatment options already available for infertility treatment in the female. FSH use for treatment of patients with normogonadotropic idiopathic infertility and oligozoospermia is still considered experimental in most countries. Recent meta-analyses have shown that FSH can significantly increase pregnancy rates in the female partners of these patients, but the effect-size is relatively low. Therefore, predictive factors for treatment success have to be identified, including FSH pharmacogenetics, to select the right normogonadotropic patients with idiopathic infertility for FSH therapy.

## Introduction

In male infertility, the indication for treatment with follicle stimulating hormone (FSH) is the induction and maintenance of spermatogenesis in patients with hypogonadotropic hypogonadism (1). As these patients are normally azoospermic without gonadotropin stimulation and during testosterone therapy, the presence of sufficiently high numbers of progressively motile and normally formed sperm in the ejaculate during exogenous gonadotropin therapy might result in the desired clinical pregnancy for many infertile couples. On an experimental basis, and in some places already in clinical routine, FSH preparations are also used for treatment of normogonadotropic infertile men with idiopathic impairment of spermatogenesis (2, 3).

The primary goal of FSH therapy in the hypogonadotropic or normogonadotropic patients is not the stimulation of testicular growth or spermatogenesis *per se*, but the induction of a pregnancy in the female partner of the infertile couple, and finally the live birth of a healthy child. This review summarizes the effects of FSH treatment on this primary clinical outcome in these two patient groups with male infertility. The administration of FSH to children or during adolescence is not the topic of this review.

## FSH therapy for Male Infertility in Patients With Hypogonadotropic Hypogonadism

In patients with hypogonadotropic hypogonadism, male infertility is due to the lack of stimulation of spermatogenesis by the gonadotropins FSH and luteinizing hormone (LH). In so-called idiopathic/isolated/congenital hypogonadotropic hypogonadism (IHH or CHH) and Kallmann syndrome, the core pathophysiological feature is the disturbed hypothalamic synthesis or secretion of gonadotropin-releasing hormone (GnRH) (4). This leads to diminished or absent LH and FSH synthesis or secretion by the unstimulated pituitary gland and finally to endocrine hypogonadism with low testosterone serum levels and infertility with azoospermia or severe oligozoospermia as the respective laboratory markers. Various other diseases including secondary GnRH deficiency lead to the same pathophysiology (4).

Other causes for hypogonadotropic hypogonadism are pituitary insufficiency due to tumors (especially makro-prolactinomas), metastases of the pituitary and the hypophyseal stalk, post-operative states, radiotherapy of the pituitary region, traumata, infections, hemochromatosis, vascular disorders, and others (4). Hypogonadotropic hypogonadism is caused by the insufficiency of the pituitary gland to secret significant levels of LH and FSH. The clinical picture in these patients is additionally influenced by possible disturbances of the other pituitary hormones.

The therapy of choice in patients with hypogonadotropic hypogonadism due to various pathophysiologic causes as mentioned above is—for most of the time of the life-span—the exogenous substitution of testosterone to maintain all androgen-dependent functions. This therapy is well established over decades, relatively convenient for the male patients and comparably inexpensive (5).

In case the patients develop the wish to have children with their female partner, the testosterone substitution therapy is no longer sufficient and has to be interrupted. The patients should then be treated with FSH preparations and in addition with a pharmacological preparation to stimulate intratesticular testosterone production by the Leydig cells. As no LH preparation is currently approved for male hypogonadotropic hypogonadism, patients are usually treated with human chorionic gonadotropin (hCG) preparations with similar, but not identical bioactivity (6). hCG has a longer elimination half-life than LH and patients can be treated effectively by two injections per week (4).

In patients with hypogonadotropic hypogonadism caused by hypothalamic disorders, exogenous pulsatile GnRH can also be used for treatment, as this will stimulate the FSH and LH secretion from the pituitary gland (4, 7). Because of the complex and time-consuming pulsatile therapy, today only few patients with hypogonadotropic hypogonadism are treated with pulsatile GnRH. Pulsatile GnRH therapy seems to have no proven advantage over FSH plus hCG therapy in patients with hypothalamic hypogonadotropic hypogonadism. However, the lack of sufficient well-designed, randomized prospective studies does not allow firm conclusions on the best therapy for infertility in these patients (8, 9).

### Early Experience With hMG in Combination With hCG Therapy

FSH has been used successfully for infertility treatment of patients with hypogonadotropic hypogonadism for more than 50 years, initially with urinary menopausal gonadotropins having FSH activity. It is worth reading the initial reports of FSH therapy in hypogonadotropic patients. MacLeod and coworkers reported on the successful therapy with urinary menopausal gonadotropins of a 37-year-old patient who underwent complete hypophysectomy in 1963 (10, 11). The patient had provided a semen sample 1 day before the hypophysectomy that showed 576 million sperm per ejaculate and quite good sperm motility and morphology. After surgery, the ejaculate quality decreased significantly and, following several weeks after hypophysectomy, the patient was unable to provide semen samples any more. Approximately 14 weeks after hypophysectomy, a bilateral testicular biopsy was performed which showed involution of spermatogenesis to the level of spermatogonia and only few areas with primary spermatocytes. One day after the first testicular biopsy, treatment with hMG (human menopausal gonadotropin originating from human urine with mainly FSH and some LH activity) was initiated in the patient, at a dose of approximately 206 I.U. per day.

After 64 days of menopausal gonadotropin treatment, another testicular biopsy only of the right testis revealed stimulated spermatogenesis, showing all stages of spermatogenesis including late elongated testicular spermatids. However, the restoration of spermatogenesis appeared only qualitatively normal, not quantitatively. As a patient was still unable to produce an ejaculate probably due to the insufficient low LH activity in the hMG preparation and therefore low testosterone serum levels, hCG therapy with 4000 I.U. on alternate days was added to stimulate testosterone production by the Leydig cells. At the same time the hMG dose of 206 I.U. was given no longer daily, but only every second day (alternating with hCG injections). With the combined therapy of hMG and hCG the patient regained the ability to produce an ejaculate that showed a total sperm count of several million with progressive sperm motility and normal sperm morphology, that were still decreased compared to the levels analyzed before hypophysectomy (11). Later the patient decided not to continue with hMG therapy and, unfortunately, no fertility data are available. However, this early comprehensive case report demonstrated clearly the principle of FSH therapy in combination with hCG for stimulation of spermatogenesis in patients with hypogonadotropic hypogonadism.

### Clinical Studies With hMG in Combination With hCG Therapy

Since then, several patients with hypogonadotropic hypogonadism were treated successfully with hMG plus hCG, for stimulation of spermatogenesis and achieving the desired pregnancy in the female partner. One of the most comprehensive studies on the treatment efficacy in patients with different etiologies for hypogonadotropic hypogonadism was published by Büchter et al. more than 20 years ago (8). This study might be regarded as one reference study for hMG treatment of these patients, as at that time hMG has been replaced more and more by highly purified or recombinant FSH preparations in the andrology clinic (12, 13). In this study by Büchter and colleagues, 21 patients with hypogonadotropic hypogonadism due to pituitary disorders were treated with hMG plus hCG. As in some of these patients more than one treatment course was performed, 30 treatment courses could be included in the study analysis. Another 18 patients with hypogonadotropic hypogonadism due to hypothalamic disorders such as Kallmann syndrome or congenital hypogonadotropic hypogonadism were treated with hMG plus hCG (18 cases, 20 treatment courses). Altogether, 31 of the 50 treatment courses with hMG plus hCG were initiated for the induction of pregnancy in the female partner and 19 of 50 courses only for the induction of spermatogenesis.

In all of the 30 treatment courses (100%) in patients with a pituitary disorder, spermatogenesis was stimulated from azoospermia to the presence of sperm in the ejaculate. In patients with a hypothalamic disorder, gonadotropin therapy induced spermatogenesis in 18 of 20 treatment courses (90%). The duration of therapy until the first detection of sperm in the ejaculate was quite variable. In the patients with a pituitary disorder, the average treatment time was 4 months (range 2–16 months). In the patients with a hypothalamic disorder, the average treatment duration was 6 months (1–18 months). The duration of time until induction of pregnancy of the female partner in patients with pituitary disorders was 10 months (2–46 months), and 8 months (1–15 months) in the patients with hypothalamic disorders. For this review article, the information on pregnancies was included that was added in proof of the publication by Büchter et al. (8). An additional pregnancy in the female partner occurred after gonadotropin treatment of one patient with pituitary disorder for 42 months as well as one patient with hypothalamic disorder treated for 48 months. Including these data, hMG plus hCG therapy in patients with a pituitary disorder resulted in 18 pregnancies in 21 treatment courses (86%) and 6 pregnancies in 10 treatment courses (60%) in patients with a hypothalamic disorder (8). Compared to other current treatments of infertility including application of assisted reproductive techniques (ART), this “causal” therapy of male infertility in patients with hypogonadotropic hypogonadism proved to be highly effective (14).

### Factors Influencing the Efficacy of Treatment

While hMG plus hCG therapy of infertile patients with hypogonadotropic hypogonadism appears to be quite successful regarding stimulation of spermatogenesis and finally clinical pregnancy induction in the female partner, the treatment might last quite long. Patients have to be informed that hormone therapy might last for several months and even years before the desired pregnancy can be achieved. Therefore, it is relevant to identify predictive factors influencing treatment efficacy (Box 1).

Box 1 **Predictive factors for treatment success.**

Clinical factors at initiation of FSH plus hCG therapy of adult male patients with hypogonadotropic hypogonadism predicting successful infertility treatment (15)

- History of normal puberty or pubertal arrest
- No history of cryptorchidism
- Higher baseline testicular volume
- Higher baseline serum levels of inhibin B

In an recent study on 51 adult patients with hypogonadotropic hypogonadism who had undergone one treatment cycle with FSH (urinary or recombinant FSH) plus hCG, those patients who had hypogonadotropic hypogonadism acquired after puberty or had a pubertal arrest showed significantly better treatment outcome (15). These patients achieved higher final bilateral testicular volume and higher final sperm concentrations compared to patients with hypogonadotropic hypogonadism manifesting before the normal onset of puberty. Most relevant, the pregnancy rate of 62% was higher in patients with post-pubertally acquired hypogonadotropic hypogonadism compared with those patients with pre-pubertally acquired hypogonadism (42%). In addition, a conception in the female partners of patients with post-pubertally acquired hypogonadotropic hypogonadism occurred significantly earlier (20.3 ± 11.5 months) than in the female partners of patients with pre-pubertally acquired hypogonadism (43.1 ± 43.8 months).

The therapeutic success was also higher in patients without previously undescended testes, in patients with higher baseline testicular volume and in patients with higher baseline inhibin B serum concentrations (15). The identification of these predictive factors is in line with various clinical studies by other study groups (16–21).

### Clinical Studies With Recombinant or Urinary FSH in Combination With hCG Therapy

No adequate large, randomized controlled trials (RCTs) have been performed to compare efficacy of recombinant or highly purified FSH with the urinary hMG preparations in males—quite in contrast to the application of FSH preparations in females for ART. From the available information, it seems that the efficacy of the various FSH preparations in male patients with hypogonadotropic hypogonadism is quite comparable, regarding stimulation of spermatogenesis and inducing the desired pregnancy in the female partner (13, 18, 20–25). Today, in Germany only recombinant FSH and no longer urinary FSH preparations are approved for this therapy in male patients.

### Common Dosing Schemes

One of the most common dosing schemes of gonadotropins in male hypogonadotropic hypogonadism is the administration of 150–225 I.U. FSH two or three times a week in combination with 1000–2500 I.U. of hCG two times per week (4). Several physicians start treatment with hCG alone for e.g., 3 months, as some patients—maybe those with some residual FSH activity—achieve stimulation of spermatogenesis by hCG alone (13, 20, 25, 26). However, the sperm concentrations seen after hCG therapy alone appear to be lower than those with the combined treatment with FSH plus hCG (27). Therefore, FSH should also be added in these hCG-treated patients at some time-point to achieve best treatment outcome. In addition, it has been shown that induction of spermatogenesis achieved by FSH plus hCG treatment in hypogonadotropic hypogonadism can be maintained qualitatively, but not quantitatively in most of the patients with hCG alone (28). On this line, a sequential therapy with 3 months treatment with FSH plus hCG alternated by hCG therapy alone for another 3 months has been proposed to reduce the relatively high costs of gonadotropin therapy (29). However, it is not known if this dosing regimen has the same high efficacy on the primary outcome clinical pregnancy rate.

The dose and injection interval of FSH might be adapted in individual hypogonadotropic patients to achieve optimal treatment outcome. The efficacy can be monitored by the increase of testicular volume, the stimulation of spermatogenesis, the serum levels of FSH achieved, the serum levels of testosterone achieved, and other factors. Unfortunately, large randomized comparative studies with different FSH preparations, different doses and different injection intervals are missing (13, 24). A retrospective study suggested that lower weekly FSH doses are sufficient to stimulate spermatogenesis and allow induction of the desired pregnancy in the female partner (24). Others have argued that the hCG dose in combination with FSH might be too high for optimal treatment effects (30). As the current FSH plus hCG dosing schemes have still the drawback of a quite long treatment duration before the desired pregnancy is achieved, it still seems rewarding to test different FSH and hCG preparations and dosing regimens by proper designed randomized controlled clinical trials to improve treatment outcome of gonadotropin therapy in male hypogonadotropic hypogonadism.

### FSH in Combination With Recombinant hCG or LH

In Germany, recombinant hCG and LH preparation are approved for reproductive hormone therapy in women. In men, so far no adequate studies have been published comparing these preparations with urinary hCG. A combination of recombinant FSH with recombinant LH or hCG in one injection pen would allow easier self-administration, more fine tuning of individual therapy, higher compliance and maybe higher treatment efficacy. In addition, it could be speculated that LH instead of hCG therapy in combination with FSH could lead to much better clinical efficacy regarding stimulation of spermatogenesis and pregnancy rate in male hypogonadotropic hypogonadism (6, 30). So far injection pens with recombinant LH are only approved for treatment of females and it is about time to provide these options also to male hypogonadotropic patients. The pharmaceutical companies should be encouraged to initiate the respective clinical studies.

### Long-Acting FSH Preparations

Another option for treatment improvement would be to use long-acting FSH analogs that are already used successfully in the fertility care of women. In a recent phase III multicenter clinical trial of corifollitropin alfa in azoospermic men with hypogonadotropic hypogonadism, it was demonstrated that administration of 150 μg of a long-acting FSH preparation given every second week leads to significant increase of testicular volume and induction of spermatogenesis, comparable to the effects seen with short-acting recombinant FSH preparations (25).

## FSH Therapy in Normogonadotropic men With Idiopathic Infertility

### Early Non-randomized Studies

As FSH therapy proved to be quite successful regarding stimulation of spermatogenesis of patients with hypogonadotropic hypogonadism and pregnancy rate in their female partners, it was tested whether this therapy can also be applied successfully in male patients with normogonadotropic idiopathic infertility. Early uncontrolled studies in these patients with hMG plus hCG therapy over a treatment period of 3 months demonstrated an increase of total sperm number in the ejaculate and also pregnancy rate in the female partners (31). The increase in pregnancy rate was especially evident in the so-called responders who were defined by an increase of sperm output of at least 25 million per ejaculate (31).

### Placebo-Controlled Randomized Studies

However, it could not be excluded that the positive FSH effects in the normogonadotropic patients in uncontrolled trials were due to the well-known regression-to-the-mean phenomenon. The efficacy of hMG plus hCG treatment for 13 weeks in normogonadotropic patients with oligozoospermia was consequently revisited in a randomized, placebo-controlled, double-blind clinical study. The positive effects seen in the uncontrolled trials could not be confirmed by this randomized study. The effects on the classical variables of ejaculate analysis according to WHO were similar in the verum and placebo group (32). However, two of 19 patients treated with hMG plus hCG achieved a pregnancy in the female partner within 2 months after cessation of treatment while no pregnancy was induced by the 20 patients treated with placebo.

Because of the low number of patients included, this randomized controlled study did not have enough power to allow conclusions about pregnancy rates. In addition, the conventional ejaculate analysis might not detect all positive effects that are relevant for fertility. FSH therapy has clear positive effects on sperm DNA condensation and fragmentation that seems to be quite relevant for fertility (2, 33–37). These aspects might be overlooked by the standard procedures for semen analysis currently recommended by WHO (38).

### Meta-Analysis of Controlled Studies

As there are no published controlled studies with sufficiently high numbers of participants yet, the effects on the pregnancy rate can only be assessed by meta-analysis. In 2013, an updated Cochrane review summarized the scientific evidence on efficacy of gonadotropin therapy in idiopathic male factor infertility to increase clinical pregnancy rate in the female partner (Table 1) (39). In this review, only RCTs with FSH/hMG alone or in combination with hCG for patients with idiopathic male factor infertility were considered that had a control group with placebo or no treatment. Finally, six RCTs were included in the analysis. The spontaneous pregnancy rate resulting from natural intercourse of 16% in the female partners of patients receiving gonadotropin treatment turned out to be significantly higher than the spontaneous pregnancy rate of 7% in the partners of men receiving placebo or no treatment (Peto odds ratio [OR] 4.94, 95% CI 2.13–11.44; 5 clinical studies; 412 participants; moderate-quality evidence). No difference was seen for pregnancy rates between the verum and control groups after additional treatment with intracytoplasmic sperm injection (ICSI) or intrauterine insemination, but the number of included patients was too low to allow final conclusions.

**Table 1:** Main results of two recent meta-analyses on pregnancy rates after FSH treatment of men with idiopathic infertility.

**Inclusion criterion**	Number of patients treated with FSH^*^	**Number of patients treated with placebo or untreated**	**Odds ratio [95% confidence interval] for spontaneous pregnancy rate^#^**	**Reference of the meta-analysis**
Randomized controlled clinical trials	201	211	4.94 [2.13–11.44]	(39)
Controlled clinical trials	384	308	4.50 [2.17–9.33]	(40)

* *In one clinical trial included in each meta-analysis (n = 19 patients in verum and n = 20 patients in control group) hMG + hCG injections were given instead of purified or recombinant FSH*.

# *Test for overall effect: Z = 3.72 (P = 0.00020) (39) and Z = 4.04 (P < 0.0001) (40)*.

Recently, another comprehensive meta-analysis (Table 1) on clinical pregnancy rate as the main outcome variable was performed by Santi et al. including all controlled clinical trials on FSH administration (including one small study with hMG plus hCG) to male partners with idiopathic infertility (40). Randomization was not an inclusion criterion for this analysis. Altogether, 15 controlled clinical trials were included with 614 men treated with FSH and 661 patients with placebo or untreated patients. Nine of the 15 studies reported on spontaneous pregnancy rate (384 FSH-treated patients, 308 control patients). The spontaneous pregnancy rate in these nine studies was significantly higher in patients treated with FSH compared to controls (OR 4.50, 95% CI 2.17–9.33). Eight studies evaluated pregnancy rate after FSH therapy and application of additional ART (322 FSH-treated patients, 275 controls). The ART pregnancy rate in this meta-analysis turned out to be significantly higher in the female partners of male patients with FSH treatment compared to controls (OR 1.60, 95% CI 1.08–2.37).

### Effect Size of FSH Therapy on Pregnancy Rate

Although these meta-analyses have demonstrated that FSH therapy in idiopathic normogonadotropic male infertility can increase clinical pregnancies in the female partners, the effect size is still relatively low. It has been calculated that 10 patients have to be treated with FSH to achieve one spontaneous pregnancy (40). Eighteen patients have to be treated with FSH to achieve one additional pregnancy after ART (40). As FSH preparations are quite expensive and many physicians involved in infertility treatment consider this effect not high enough, FSH treatment in idiopathic male infertility is so far not part of the routine treatment regime and not covered by insurance companies in many countries (3).

### Selecting the Right Normogonadotropic Patient for FSH Therapy

To overcome these shortcomings, it is mandatory to select the right patients for FSH therapy. The evidence on FSH therapy in normogonadotropic patients is so far restricted to idiopathic infertility, which means that no identifiable, generally accepted cause for male infertility could be detected. Therefore, all male patients have to have a proper diagnostic andrological work-up before initiating any FSH therapy, and also the female partner needs to have a proper diagnostic gynecological work-up! Normogonadotropic male patients with identifiable and possibly treatable causes for male infertility (e.g., obstruction) should not be treated with FSH. Obviously, male patients with elevated bioactive FSH serum levels should not receive FSH therapy. It has been suggested not to provide FSH treatment to infertile patients with hypospermatogenesis associated with maturational disturbances at the spermatid level (2).

In addition to these factors, FSH therapy in normozoospermic infertile patients might be improved by optimizing the FSH dose, injection interval and especially treatment duration (1). In a recent prospective, double-blind, placebo-controlled clinical study in China including 354 patients with idiopathic oligozoospermia, the best results were seen in patients treated with the highest FSH dose of 300 I.U. every second day and for the longest treatment duration (maximal treatment duration in this study was 5 months) (41). Using the knowledge from the gonadotropin therapy in hypogonadotropic patients, it could be speculated that longer treatment of normogonadotropic patients would also improve pregnancy rates in the female partner (8).

One other promising approach to improve treatment outcome might be the application of FSH pharmacogenetics. Single nucleic polymorphism (SNP) p.N680S in exon 10 of the FSH receptor gene (*FSHR*) has been shown to influence the ovarian response during controlled ovarian stimulation (42). Therefore, it was logical to test the effect of p.N680S also regarding FSH therapy for normogonadotropic patients with idiopathic infertility (34). In this study, the primary outcome variable was DNA fragmentation index (DFI) of sperm in the ejaculate (36, 37). It could be shown that total DFI decreased significantly from baseline to the end of the study—a surrogate effect indicating improved fertility—in male patients with the p.N680S homozygous N polymorphism, but not in the patients with p.N680S homozygous S polymorphism of the FSH receptor. These findings indicate that a selection of the right normogonadotropic patients for FSH therapy might be possible, and probably different treatment regimens could be used for different patients groups.

Several pharmacogenetic studies have been performed in normogonadotropic men over the last years that tested SNPs of the FSH beta subunit gene (*FSHB*), SNPs of the *FSHR*, or combinations thereof (34, 35, 43, 44). These trials have been reviewed comprehensibly by Schubert and co-workers in this Research Topic of Frontiers in Endocrinology (45). Unfortunately, these clinical trials come to quite divergent study results, probably due to study design, inclusion criteria, and as one of the main factors FSH doses and injection intervals. However, only large, placebo-controlled, randomized multicentre studies with pregnancy rate—and not any surrogate marker—as the primary outcome variable will finally allow a conclusion on the value of FSH pharmacogenetics to select the right normogonadotropic patients with idiopathic infertility for FSH therapy.

### FSH Therapy in Patients With Failed TESE

FSH therapy is currently suggested by different clinicians for patients with idiopathic azoospermia after failed testicular sperm extraction (TESE) (46–48). So far, these studies are case reports or have low patient numbers and do not include a randomized control group. Therefore, no firm conclusion on the efficacy of FSH therapy in these patients with idiopathic azoospermia is possible, yet.

## Perspectives

Although ART treatment is quite effective for infertile couples regarding the desired clinical pregnancy and the birth of a healthy child, there is a growing demand from patients and health authorities to apply effective causal therapies for the infertile male whenever possible and meaningful. Therefore, a systematic andrological examination of the male partner of the infertile couple should always be performed, even when some gynecological reasons for infertility have already been identified. In Germany, the thorough andrological work-up is now mandatory before any ART therapy can be initiated (49).

Hypogonadotropic hypogonadism is a good example how causal hormone therapy of male infertility can be applied with high clinical efficacy regarding induction of pregnancy in the female partner. It remains to be seen if FSH therapy will also be useful and generally accepted for treatment of male infertility in normogonadotropic patients with idiopathic impairment of spermatogenesis.

Without question, more well-designed, prospective randomized studies are needed to identify the best FSH treatment for the infertile male patient. All relevant players in the healthcare system should be stimulated to provide the respective resources for optimizing treatment outcome of male infertility—in the male!

## Author Contributions

All work for this article was done by HB.

### Conflict of Interest Statement

The author declares that the research was conducted in the absence of any commercial or financial relationships that could be construed as a potential conflict of interest.
